# New Frontiers in Stem Cell Research and Translational Approaches

**DOI:** 10.3390/biology9010011

**Published:** 2020-01-04

**Authors:** Nicola Alessio, Dario Siniscalco, Gianfranco Peluso, Umberto Galderisi

**Affiliations:** 1 Department of Experimental Medicine, Division of Molecular Biology, Biotechnology and Histology. University of Campania “Luigi Vanvitelli”, via S. Maria di Costantinopoli 16, 80138 Naples, Italy; nicola.alessio@yahoo.it (N.A.); umberto.galderisi@unicampania.it (U.G.); 2 Research Institute on Terrestrial Ecosystems (IRET), National Research Council of Italy, (CNR), via P. Castellino 111, 80131 Naples, Italy; gianfranco.peluso@cnr.it

**Keywords:** stem cells, translational approach, regenerative medicine, cellular therapy

## Abstract

Stem cell biology represents a challenging research area with a huge potential translational approach. This review focuses on the most recent findings on stem cell basics and clinics in several fields of research, as final outcome of the 10th conference held by Stem Cell Research Italy (SCR Italy) in Naples, Italy in June 2019. Current state-of-the-art and novel findings on stem cell research are discussed, bringing together basic and applied research with the newest insights in stem cell therapy.

## 1. Introduction

Today, it is well-known that stem cell therapy could offer great promise for molecular and regenerative medicine [1]. Due to the wide availability of several stem cell subtypes, the clinical application of cellular therapies is a real tool to face some untreatable human diseases. To date, more than 30 cell subtypes have been discovered that show the capacity to restore damaged tissues [2]. However, it should be pointed out that further studies on stem cell biology are needed before using them in clinical applications [1].

The focus for this review is on the most recent findings in stem cell basics and clinics across several fields of research, as the final outcome of the 10th conference held by Stem Cell Research Italy (SCR Italy) at Naples, Italy in June 2019. Indeed, the aim of this conference was to exchange information regarding today’s state-of-the-art and novel findings in stem cell research, bringing together basic and applied research with the newest insights in stem cell therapy. The conference also provided an excellent opportunity for young stem cell researchers to show their findings, and to have discussions with internationally recognized stem cell experts. The conference was held at the Department of Experimental Medicine of the University of Campania “Luigi Vanitelli” in Naples. Naples is the regional capital of Campania and the third largest municipality in Italy, after Rome and Milan, and one of the most densely populated cities in Europe.

## 2. Stem Cells and Regenerative Medicine

The speaker for the first key lecture was Dr. Graziella Pellegrini (University of Modena and Reggio Emilia, Modena, Italy) who provided a detailed explanation of regenerative medicine, which has generated many efforts to explore new therapeutic potentials of both somatic and pluripotent stem cells with several possibilities envisaged for therapeutic applications. In detail, she spoke about hematopoietic and epithelial cells that are extensively adopted for tissue regeneration, due to their high proliferative capacity and accessibility. Discoveries and recent developments in cell-based therapy for ocular burns were illustrated, providing support for improving and standardizing the cure for this disabling eye disease, which causes depletion of limbal stem cells. How biopsies have been taken from a healthy eye or from another autologous source, as oral mucosa was explained [3]. The key point of this lecture was that only a few of these therapies overcome the hurdles related to medicinal product regulation and become available to patients, but the combined use of cell and gene therapy represents a further scientific approach for the treatment of congenital diseases. This approach has been proven on hematopoietic cells and has recently been established using genetically modified epidermal cells for life-saving treatment on severe genetic diseases, such as epidermolysis bullosa.

## 3. Stem Cells and Muscle Diseases

The second speaker, Dr. Antonio Musarò (Sapienza University of Rome, Rome, Italy), opened the first session of the conference “Stem cells and muscle diseases” and spoke about how muscle regeneration is an important homeostatic process of adult skeletal muscle. In detail, the focus was on the role of the cellular niche that could be responsible for the maintenance of the stem cell pool under steady-state conditions, as well as guiding stem cell activation and differentiation when regenerative signals are provided. How the loss of homeostatic inputs from the niche, as observed under pathological conditions, can deregulate stem cell physiology, critically affecting the ability of muscle tissue to efficiently regenerate and regain functional integrity after damage were illustrated. Thus, the stem cell niche is not only an anatomical compartment, but a complex, integrated network of both cellular and acellular (as cytokines and growth factors) components that provide chemical signals influencing stem cells and muscle homeostasis [4].

Similar to the previous speaker, the third speaker, Dr. Ombretta Guardiola (Institute of Genetics and Biophysics, National Research Council (CNR), Naples, Italy), spoke on the quiescent skeletal muscle stem cell-satellite cell (SC) heterogeneity. Recent data was illustrated that point to a key role of the extracellular membrane protein Cripto. Dr. Ombretta showed a non-homogeneous expression of Cripto in the SC pool and distinguished two proliferating subpopulations based on Cripto expression levels (CriptoHigh and CriptoLow) [5]. These cellular populations display different functional and molecular characteristics; CriptoLow cells show less internal cell complexity, lower mitochondrial activity, and a delayed cell cycle progression as compared with CriptoHigh cells that, on the contrary, are more primed for myogenic commitment. Indeed, cells derived from CriptoHigh mainly generate differentiating cells (Pax7neg) in vitro and a low number of undifferentiated (likely self-renewing) Pax7pos as compared with CriptoLow cells. However, Pax7pos cells derived from both populations re-establish the in vitro heterogeneous expression of Cripto, indicating that the distinction between the two subpopulations is dynamic and is subject to the perturbations that occur in the whole population, by inducing fluctuations in Cripto expression. Finally, Cripto-cKO SCs phenotype mimics that of wild-type CriptoLow cells.

The first session ended, leaving ample time for young researchers’ presentations. The first was Dr. Edoardo Maghin (Fondazione Istituto di Ricerca Pediatrica Città della Speranza, Padua, Italy) who spoke on congenital diaphragmatic hernia (CDH), which requires early surgical intervention, with the application of artificial patches to repair large defects. Unfortunately, these materials face major issues, such as the lack of growth and integration with the surrounding muscle tissue, leading to an associated high rate of hernia. Recently, Dr. Maghin focused his attention on the characterization of the extracellular matrix (ECM) obtained from decellularized mouse diaphragm and showed that the use of a muscle-specific decellularized scaffold as a substitute for currently used synthetic materials allows new blood vessel growth, long-term muscle regeneration, and reinnervation inside the dECM, improving diaphragm performance and functional recovery [6]. Despite the positive clinical and functional outcomes, it was reported that the dECM patches did not activate complete defect regeneration and that this could be exceeded with the recellularization approach and the in vitro generation of tissue-like construct before in vivo applications. It was demonstrated that dECM was a suitable scaffold for the growth and in vitro differentiation of human muscle precursor cells (hMPCs), allowing the generation of a functioning three-dimensional skeletal muscle structure, containing both muscle stem cells and differentiated myotubes. One of the major challenges for in vitro regeneration of the skeletal muscle is the degree of the fiber organization and alignment inside the dECM, and therefore the newest studies on the bioreactor platform that exploits numerical modeling based on the finite element method (FEM) approach to estimate correct structural behavior were shown. After pilot experiments, in which the mechanical radial strain was applied individually to the dECM and hMPCs, recellularized diaphragmatic-like constructs were cultivated in dynamic conditions for 21 days, demonstrating the maintenance of proliferating cell pool, but also an increased myotubes alignment and maturation.

Dr. Cecilia Mei (University of Rieti, Rieti, Italy) demonstrated that mesenchymal-stromal stem cells (MSCs) isolated from dental pulp (DPSCs) show a high proliferative potential [7]. In vitro DPSCs seem to stabilize endothelial tubes behaving similar to pericyte-like cells. Specifically, how culture conditions can modulate expression of pericytic markers in DPSCs and their capacity to stabilize endothelial tubes in vitro were evaluated. When cultured in an endothelial medium (EGM-2), DPSCs significantly upregulated NG2 expression, and partially alfa-smooth muscle actin (α-SMA). The ulting cellular population conserved the stem cell marker CD73 expression, but it was negative for calponin and endothelial markers. EGM-2-conditioned DPSCs (E-DPSCs) showed a higher sprouting ability in the EC-matrix and efficiently associated with human umbilical vein endothelial cells (HUVECs), allowing the partial retention of endothelial tubes for several days. The bFGF factor, from among the factors contained in EGM-2, was identified as the main factor responsible for NG2 upregulation and long-term stabilization of endothelial tubes. In conclusion, DPSCs seem to represent an effective source of pericytes and the appropriate culture conditions could result in a population with a promising ability to stabilize vessels and promote vascular maturation [7].

The last young researcher who presented at this first session was Dr. Fabiana Geraci (University of Palermo, Palermo, Italy), who has demonstrated that mesoangioblast extracellular vesicles (EVs) modified the expression pattern of cytokines and chemokines released into the extracellular medium by macrophagic RAW264.7 cells. The EVs increase the expression of several cytokines. In particular, upregulation of cytokines involved in an anti-inflammatory response (e.g., IL-10, IL-6, and IL-1ra) and downregulation in some inflammatory cytokines (e.g., IFN-γ, IL-12 p40, and IL-23) has been shown. These data suggest that EVs polarize macrophages towards an anti-inflammatory M2 phenotype. In order to better characterize the EV influence on macrophages, proliferation assays were carried out and showed that A6-EV treated cells have a higher proliferation index than that of untreated cells. Furthermore, it was demonstrated that wound-healing assays with EVs increase RAW264.7 migration capability. The A6-EV cells are also able to increase the phagocytic activity of macrophages. The literature data demonstrate that the heat shock protein Hsp70 is responsible for macrophagic activity increase and, as already demonstrated in a previous paper, that A6-EVs express Hsp70 on their surface [8]. For this reason, a phagocytosis assay was performed in the presence of neutralizing antibodies for Hsp70 and for two of its receptors (i.e., TLR2 and TLR4), the competitor for the receptor CD91, and in the presence of exogenous Hsp70. The data demonstrated that A6-EVs increase phagocytosis through Hsp70 and its receptors, in particular, TLR4, as well as showed preliminary results of A6-EV interaction with Jurkat cells. No effects were observed on Jurkat proliferation ability, but it seems that EVs induce Jurkat cells to increase the expression of the surface marker, CD193, a protein also expressed on Th2 cells, suggesting a possible switch in the phenotype of T cells towards a Th2 phenotype.

A new session entitled “From basic to applied research with stem cells” introduced a new day of conference work. The first speaker of the new session was Dr. Veronica Albertini (SSCB SwissStem Cells Biotech, Vacallo, Swiss), who spoke about the good manufacturing practice (GMP) of hematopoietic progenitor cells from umbilical cord blood as possible applications in regenerative medicine. Because cells used for transplantation in humans are considered advanced medicinal products, they require production and quality controls in compliance with GMP regulations in order to achieve standardization of the methods of cell productions. The GMP process is mandatory for new incoming cell therapies, i.e., cord blood stem cell infusions. The GMP guidelines for advance therapies medicinal products state that the production can be performed in a dedicated clean room (with laboratories of grade D, C, and B) or in an isolator, a positive pressure isolator, placed in a background clean area of grade D laboratories. Isolators should be introduced after appropriate validation, considering all critical factors of isolator technology, i.e., the quality of the air inside and outside (background) the isolator, disinfection regime of the isolator, the transfer process, and the isolator’s integrity. As part of the presentation, two possible choices were compared, a clean room versus isolator technology, in terms of the time of implementation, initial costs, maintenance, and ease-to-use for the lab personnel.

The next speaker was Dr. Letizia Penolazzi (University of Ferrara, Ferrara, Italy) who spoke about intervertebral disc (IVD) degeneration that can occur in any area of the spine, for different reasons (trauma, overuse injuries, ageing, and genetic factors). It is a major cause of lower back pain and a leading cause of disability worldwide. Little is known about the molecular regulators that drive cellular changes and can shift the balance between self-repair capacity and microenvironment deterioration. Dr. Penolazzi focused on the signaling mediated by the TRPS1 transcription factor and found that the reciprocal regulation between TRPS1 and a potent antichondrogenic microRNA, miR-221, seems to be of crucial importance for the maintenance of the IVD homeostasis and disc cell functions [9]. This study originated from the following two key observations: (1) Highly degenerated IVD expressed low levels of TRPS1, on the contrary, high-level expression of TRPS1 was significantly associated with lower pathological stage and (2) IVD cells benefit from TRPS1 overexpression that accompanies the loss of miR-221. The validation of the relationship between these two molecules was the results of Luciferase reporter assay and chromatin immunoprecipitation experiments on human primary IVD cells, demonstrating that miR-221 directly targets 3′-UTR of *TRPS1* gene, and TRPS1 is in vivo recruited at the miR-221 promoter region. This hypothesis is strengthened by the fact that the chondroprotective effect of TRPS1 overexpression is associated with expression increase of standard chondrogenic markers, such as COL2A1, SOX9, and ACAN that are also required for discogenic differentiation, and two important molecules in the tissue regeneration and repair, SOD2 and SOX2. The SOD2 molecule is the major mitochondrial antioxidant protein and plays a key role in regulating oxidative stress resistance. Therefore, an increase in the expression of this protein can support the ability of IVD cells to neutralize reactive oxygen species and decrease oxidative damage. Regarding the SOX2 molecule, a documented stemness regulator, the data suggest that high levels of TRPS1 can support the action of the resident stem cell population by increasing their regenerative potential, and consequently, could positively impact IVD regeneration and tissue engineering strategies. Therefore, these results provide new targets for the treatment of disc degeneration, suggesting that the hostile degenerated IVD microenvironment could be counteracted by regenerative and reparative strategies aimed at maintaining or stimulating high levels of TRPS1 through inhibition of one of its negative regulators such as miR-221 [9].

The next speaker, Dr. Simone Pacini (University of Siena, Siena, Italy), spoke about a clinical-grade and selective culture method that his team developed to isolate mesangiogenic progenitor cells (MPCs) with a high grade of purity from human bone marrow. MPCs are characterized by a lack of MSC markers, specific integrin profile, and phenotype that include CD31 and, surprisingly, CD64 and CD45 [10]. These cells showed both mesogenic and angiogenic potential in vitro. Mesengenic differentiation protocol has been established in chemically defined conditions and, more recently, the angiogenic potential has also beenclearly demonstrated in vivo, applying MPC constructs on chicken chorioallantoic membrane. Surprisingly, the ex vivo precursor of MPCs in hBM has been identified in the CD45dimCD64brightCD31brightCD14neg population with a morphology resembling monoblasts. Dr Pacini explained that, in proof-of-concept experiments, MPCs have been transplanted in nude mice, by subcutaneous injection, as single cell suspensions or three-dimensional (3D) spheroids after angiogenic stimulation. Ossicles resulted from the transplantation of 3D spheroids showed heterogeneous tissues composed of the unmineralized osteoid rich collagen type I fibers and almost acellular “interstitial” space rich in mature adipocytes and perfused sinusoid-like vessel microvessels, strongly resembling the bone marrow cavity microanatomy. Because of their peculiar differentiation properties and clinical-grade isolating method, MPCs could represent a new tool for the implementation of cell-based medicinal products (CBMPs) applicable for skeletal tissue regeneration, as these cells coupled neoangiogenesis and endochondral ossification. The coexistence of mesogenic and angiogenic potential in MPCs could significantly improve the regeneration potential of new therapeutic approaches involving these interesting cells.

Dr. Paolo Netti (University of Naples Federico II, Napoli, Italy) talked about the precise and quantitative control of stem cells. Today, the processes for controlling stem cell fate, using chemicals or transfections, strongly limit its clinical implementation. Literature data has demonstrated that the biophysical signals of culturing substrate are effective regulators of stem cell functions, as proliferation and differentiation. Deciphering cell-material crosstalk at the interface could help to disclose mechanisms by which cells sense biochemical, topographic, and mechanical signals and translate them into commands that regulate activity and fate. Results gathered lately by this research group, demonstrated that in vitro controlling cell adhesion-mediated pathways, could lead to a deterministic control of stem cell fate and launch a morphogenetic program towards the development of in vitro 3D thick human tissue [11]. Indeed, by using nanopattern material morphophysical cues it is possible to control stem cell fate and functions via focal-adhesion mediated cell-cytoskeleton crosstalk. Materials engineering, functionalization methods, and nanofabrication technologies have provided researchers with artificial alternatives to conventional rigid plates or glass, and in this way they can effectively mimic the native microenvironment. These results highlight the importance of patterned morphophysical signals on supporting materials in providing a powerful instructive microenvironment capable of triggering and guiding even complex cell processes without the use of exogenous chemicals or gene transfection.

Dr. Marina Melone (University of Campania, Napoli, Italy) spoke about the neurodegenerative diseases which are mainly characterized by the gradual dysfunction and progressive loss of neurons in the brain or spinal cord and loss of stem cells function. Presently, there are no effective treatments that are able to halt the progression of many neurodegenerative diseases. Although no effective cures are available, recent findings speculate that potential treatments for these disorders could be aimed at rescuing impaired plasticity of the brain. Over the last decade, stem cell therapy has been proposed as a treatment option for neurodegenerative and neurodevelopmental diseases, that are characterized by an abnormal development of the central nervous system, leading to a myriad of symptoms. In addition, the plasticity of the brain has been severely impaired. These neurodevelopmental disorders include autism spectrum disorders (ASDs), Down syndrome (DS), fragile X syndrome (FXS), and Rett syndrome (RTT). Although no effective cures are available, recent findings speculate that potential treatments for these disorders could be aimed at rescuing impaired plasticity of the brain. In particular, on the one hand, stem cell-based therapies for neurodegenerative diseases aim at halting neuronal deterioration by providing local support for damaged tissue. On the other hand, stem cell-based therapies for neurodevelopmental diseases aim to rescue the impaired molecular, cellular or tissue plasticity of the brain. To date, several stem cell-based clinical trials have been completed for these diseases, but they have not yielded satisfactory results leading to considerable clinical improvements. Although stem cell-based therapy seems to be a promising approach, much research remains to be completed for maximum optimization of its efficacy in neurodegenerative and neurodevelopmental disease treatment.

The second session also reserved ample time to young researchers. Among these, the first speaker was Dr. Alice Masserdotti (University Cattolica del Sacro Cuore, Rome, Italy) who spoke about amniotic mesenchymal stromal cells (hAMSC) isolated from human term placenta and hAMSC-derived conditioned medium (CM-hAMSC) that showed anti-inflammatory and antiproliferative properties, affecting cells of the innate and adaptive immune systems (Th1, Th17, dendritic cells, monocytes, and macrophages), and supporting the expansion of regulatory T cells and anti-inflammatory macrophages (M2). Although the precise factors and mechanisms responsible for the immunoregulatory role of hAMSC remain unknown, Dr. Masserdotti has shown that prostanoids could be one of the key effector molecules by demonstrating that the antiproliferative effect was partially reverted using CM-hAMSC without prostanoids. The purpose of this study was to investigate the effect of hAMSC and CM-hAMSC on B cell proliferation and subsets and to evaluate the possible role of prostanoids. For this reason, PBMC and B cells, isolated from healthy donors with CpG-ODN, were stimulated in the presence or absence of hAMSC or CM-hAMSC. Similar experiments were performed using CM-hAMSC without prostanoids, produced in the presence of a cyclooxygenase inhibitor (indomethacin). The data showed that hAMSC and CM-hAMSC significantly inhibited the proliferation of stimulated B cells as compared with the control. Moreover, considering the B cell composition (CD19+), treatment with CM-hAMSC mostly affected antibody-secreting cells (ASC, CD27high CD38high). CpG-ODN stimulation of B cells led to an increase of ASC numbers, and in particular, induced the maturation of plasmablasts (CD27high CD38high CD138-) into terminally differentiated plasma cells (CD27high CD38high CD138+). Treatment with CM-hAMSC not only reduced ASC formation, but also blocked the differentiation at the plasmablast level. The analysis of the impact of prostanoids on anti-inflammatory and antiproliferative properties of CM-hAMSC demonstrated that the use of indomethacin partially reverted the antiproliferative capacity of CM-hAMSC and ASC formation, indicating that prostanoids interfere with these processes. However, the resulting ASC were mainly plasmablasts, suggesting that prostanoids are not involved in the terminal maturation into plasma cells.

The second young researcher of this session was Dr. Marcella La Noce (University of Campania, Napoli, Italy) who spoke about adult stem cells isolated from the dental pulp which is an interesting source of postnatal progenitor cells and stem cells [12]. The aim of this study was to evaluate the osteogenic differentiation of dental pulp stem cells (DPSCs) treated with the following different histone deacetylase inhibitors (HDACi): Valproic acid (VPA), entinostat (MS275), trichostatin A (TSA), and vorinostat (SAHA). First, the effects of these inhibitors on cell proliferation, viability, bone-associated gene expression, and matrix mineralization were demonstrated. After treatments, VPA was the drug that induced osteogenic differentiation; at low concentration, it was sufficient to significantly enhance matrix mineralization by increasing osteopontin and bone sialoprotein expression. In contrast, osteocalcin levels were decreased, an effect induced at the transcriptional level, and were strongly correlated with inhibition of HDAC2. Indeed, HDAC2 silencing with shRNA produced a similar effect to that of VPA treatment on the expression of osteoblast-related markers. Furthermore, in vitro data were confirmed by in vivo studies. Hematoxylin and eosin and Alizarin Red stainings highlighted a strong trend of VPA-treated cells to form a dense connective tissue. Within this tissue, a rather large portion is visible, that is even more dense and organized, highly colorable (very eosinophilic), similar to a bone ossification center. Immunofluorescence analysis to evaluate the expression of OPN and OC confirmed the results obtained in vitro and in vivo. Consequently, the molecular mechanisms by which VPA exerts those effects on MSCs were investigated and a direct correlation between the glucocorticoid receptor (GR) and HDAC2 inhibition exerted by VPA treatment was identified. Furthermore, using co-immunoprecipitation analysis, for the first time, a binding between GR and HDAC2 in the cytoplasm was shown. Additionally, chromatin immunoprecipitation (ChIP) assays confirmed the role of GR in OC downregulation, showing recruitment of GR to the nGRE element in the OC promoter. These results open new directions in targeted therapies and offer new insights into the regulation of MSCs fate determination [12].

The third speaker among the young researchers was Dr. Sara Cruciani (University of Sassari, Sassari, Italy) who spoke about nutraceuticals, as pharmaceutical preparation or food supplements, that can influence epigenetic modulators, inducing chromatin remodeling activating specific signaling pathways influencing cell behavior. Berries of *Myrtus communis*, a typical Mediterranean shrub, are rich in phytochemicals, such as tannins and flavonoids, and are largely known for the liqueur production [13]. After a hydroalcoholic infusion, myrtle berry can be pressed, freeze-dried to remove water and, then, lyophilized. Thus, the obtained extracts, which are residuals of the industrial production, are used to test their biological activity. This study aimed to evaluate the properties of the extracts from Myrtus berries pulp and seeds at a concentration of 0.5 mg/mL, after 12, 24, and 48 h, on human adipose-derived stem cells (ADSCs), isolated from male and female patients after surgery processes. Cytotoxicity of the extracts was evaluated using MTT assay in cells cultured in the presence or absence of the extracts under H_2_O_2_-induced oxidative stress. Then, ROS production and the expression of inflammatory cytokines, pluripotency-related genes, and sirtuin-dependent epigenetic changes were evaluated. Senescent cells were determined by SA-β-Gal staining. The results obtained showed that Myrtus is able to exert antioxidant and anti-inflammatory activity, significantly reducing nitric oxide production and expression of IL-6. Moreover, pluripotency-related genes (OCT-4, SOX2, and NANOG) were upregulated in cells treated with the extracts as compared with the control untreated cells. Cells treated with the extracts exhibited an upregulation of *SIRT1* expression and a reduced number of senescent cells as compared with the control untreated H_2_O_2_-stressed cells. In conclusion, the results showed that Myrtus products from industrial waste maintain antioxidant, anti-aging, and anti-inflammatory activities, preserving ADSCs pluripotency and protecting them from oxidative stress damages. Taken together, these findings disclose novel approaches for food supplements as pharmaceutical preparations, in regenerative medicine and ameliorating the evolution of several diseases [13].

The next young researcher of this second session was Dr. Monica Mattioli (University of Milan, Milan, Italy) speaking about pharmacological modulation of neural stem cell (NSC) proliferation and differentiation into neurons as an attractive strategy for treating neurodegenerative diseases [14]. NSC neuronal differentiation requires a metabolic shift towards oxidative phosphorylation. To maximally favor and increase oxidative metabolism and mitochondrial function, Dr. Mattiolithis designed a formulation composed of essential amino acids, TCA cycle precursors, and cofactors and explored its effect on NSC neuronal in vitro differentiation and found that the treatment greatly increased mitochondrial distribution, oxidative phosphorylation, and ATP production in differentiating neurons derived both from murine NSCs and from human induced pluripotent stem cells (iPSCs). The enhanced mitochondrial function was associated with increased neuronal differentiation of treated cells. Morphological analysis revealed an increase of the following: (i) The total dendritic length, (ii) the mean number of branches, and (iii)the number and maturation of the dendritic spines. Furthermore, neuronal spines in the treated neurons appear more stable with a stubby and “mushroom” phenotype and with increased expression of molecules involved in synapse formation. At the molecular level, protein expressions indicated that treated neurons modify their mitochondrial dynamics increasing the mitochondrial fusion. Moreover, it was found that treated neurons highly activate mTOR dependent p70S6K and increase anabolic activity (synthesis of intermediates, including proteins, fatty acids and membranes). Global transcriptomic analysis (RNA sequencing) further revealed that treated neurons strongly induce Nrf2-mediated gene expression that greatly increased glutathione metabolism and functionally improved ROS scavenging mechanisms. In conclusion, these results indicated that this specific treatment boosts the metabolic switch toward oxidative metabolism, increases mTOR, ROS defenses, and enhances neuronal differentiation. Rewiring neuronal metabolism towards oxidative metabolism in neurodegenerative diseases, including stroke, brain, and spinal cord injury could be an effective therapy to improve neural function.

The sixth young researcher for this session was Dr. Valeria Pizzuti (University of Bologna, Bologna, Italy) who spoke on how in vitro cell culturing can modify membrane lipidomics as compared with in vivo conditions. These alterations have consequences on cell structural, biophysical, and functional characteristics, and they do not guarantee the full efficiency of most cell processes. Commonly used culture media often have deficiencies in lipid components, which are essential for maintaining proper composition and structure of the membranes. The aim of this study was to add a custom lipid supplement to culture conditions to evaluate if it was able to maintain a membrane lipidomic signature more similar to the in vivo one. In particular, the addition of a custom lipid supplement named Refeed® on cultured human amniotic membrane epithelial cells (hAECs) was tested showing that membrane lipidomic signature of hAECs is altered by in vitro cultivation. The addition of a tailored lipid supplement in the culture medium is associated with a membrane composition closer to that found in vivo. The optimization of the culture conditions appears to be associated with increased migratory capacity and the late development of senescence characteristics. Further studies have to be performed to evaluate the expression of proteins involved in p21 and p16 pathways. Understanding the effects of changes in membrane lipidomic signature could have useful implications in the development of in vitro models closer to in vivo complexity and in cell-based therapeutic approaches.

The last young researcher of the second session was Dr. Gloria Bedini (University of Milan-Bicocca, Milan, Italy) who spoke about Shwachman–Diamond syndrome, an autosomal-recessive inherited bone marrow failure disorder with defective angiogenesis [15]. Shwachman–Diamond syndrome (SDS, MIM 260400) is a rare autosomal recessive bone marrow (BM) failure disorder characterized by neutropenia (found in 88% to 100% of patients), exocrine pancreatic insufficiency, and skeletal abnormalities. Pancytopenia occurs in 10% to 65% of cases and, similar to other BM syndromes, SDS patients show an increased risk of myelodysplastic syndrome and malignant transformation. Shwachman–Bodian–Diamond Syndrome (SBDS) mutations have been identified in approximately 90% of these patients. Proteomic data implicated SBDS in RNA metabolism, ribosomal function, and stromal microenvironment. The key elements of the BM stroma are mesenchymal stromal cells (MSCs), a unique archetype of multipotent somatic cells that are able to maintain and support the formation of hematopoietic stem cell niche. This research group has previously demonstrated that SDS-MSCs were similar to healthy donor (HD)-MSCs in terms of morphology, growth properties, surface epitopes, and differentiation ability. However, gene expression analysis showed that several genes involved in hematopoietic cell development, lymphopoiesis and neoplastic progression were differently expressed between SDS-MSCs vs. HD-MSCs. In light of the pivotal role of MSCs in regulating the hematopoietic compartment of the BM niche, it was demonstrated that in vivo implanted semi-cartilaginous pellets derived from SDS-MSCs did not generate a very incomplete niche with either vascularization or hematopoietic cell invasion. Moreover, by an in vitro angiogenesis assay it was demonstrated that SDS-MSCs showed a defective ability to form correct tube networks after specific angiogenic stimuli, displaying a marked decrease in VEGFA expression. Therefore, to deeply investigate the angiogenic changes underlying this vascular instability and to evaluate a possible link between hematological abnormalities observed in SDS patients and defective angiogenesis, the in vitro results were extended on a larger cohort of SDS patients and HD-MSCs demonstrating that none of the SDS-MSCs was able to recreate a well-defined tubular structure (SDS = 0/10 and HD = 8/12). Specifically, SDS-MSCs showed a clear cell detachment followed by a reduced number of master junction (*p* = 0.006), meshes (*p* = 0.007), and branching intervals (*p* < 0.001) in the vascular network as compared with HD-MSCs. From the molecular analysis, it was found that *VEGFA* expression was upregulated in both SDS and HD-MSCs; however, this increase was lower in SDS-MSCs as compared with HD-MSCs (*p* = 0.015). VEGFA can induce VEGF receptor-negative MSCs to migrate and proliferate, by binding and activating PDGFRs. MSCs in vitro have a high cell surface PDGFRα:PDGFRβ ratio, with abundant PDGFRα appearing to be a characteristic of undifferentiated MSCs. Surprisingly, from the molecular analysis, it was found that PDGFRβ is more upregulated in SDS vs. HD-MSCs (*p* = 0.049). These data were also confirmed by the downregulation of the PDGFR ratio, further sustaining the specific upregulation of PDGFRβ in SDS-MSCs.

The third section entitled “Stem cells and cancer” was opened by Dr. Persio Dello Sbarba (University of Firenze, Florence, Italy) who spoked about the relationship of the maintenance of stem cell potential of hematopoietic stem cells to specific environmental conditions in bone marrow [16]. These conditions, referred to as the stem cell niche (SCN), depend on the action of a number of cellular and molecular players, the latter including critical levels of nutrients, oxygen and glucose in particular, involved in energy production. These factors undoubtedly also act in leukemias, due to the strict analogy between their hierarchical structure and that of normal hematopoietic cell populations. On the basis of this, Dr. Sbarba undertook the characterization of the response of leukemia stem cells (LSC) of chronic myeloid leukemia (CML) to nutritional constraints and found that the incubation of CML cells in low oxygen, a physiological feature of SCN in vivo, time-dependently suppresses BCR/Ablprotein, the product of the fusion oncogene responsible for CML pathogenesis, but not BCR/AblmRNA. Thus, on the one hand, an LSC subset exists which is capable of persisting independent of BCR/Abl signaling yet remaining genetically leukemic. These LSC are completely refractory to BCR/Abl-active tyrosine kinase inhibitors (TKi) used for CML therapy, obviously due to the lack of their molecular target. On the other hand, a subset of BCR/Ablprotein-expressing cells also exists which exhibits LSC properties. It was proposed that the latter LSC subset is optimized to drive the expansion of leukemic cell population under nutritionally permissive environmental conditions, while the BCR/Ablprotein-negative LSC are responsible for the long-term maintenance of therapy-resistant minimal residual disease (MRD) and the related risk of relapse of CML following successful induction of remission. In this model, a relapse of disease would occur when the LSC adapted to energy shortage, following the establishment of permissive conditions (restored glucose supply), induced to express BCR/Ablprotein or to generate a BCR/Ablprotein-expressing LSC progeny, and thus lead to the triggering of clonal expansion. Two different LSC subsets seem to suit, respectively, the two alternative routes suggested to generate cancer stem cells, that is, the oncogenic transformation of a normal (self-renewing) stem cell or the oncogenic staminalization (acquisition of self-renewal) of a normal progenitor cell. All the above led to envising a two-tier model for SCN of CML which included the following: (1) A niche core where glucose is absent but other nutrients are available and metabolized, and where LSC sustaining MRD reside; (2) a highly glycolytic niche periphery hosting LSC ready to drive clonal expansion; and (3) a sort of “metabolic symbiosis” between the two areas, driving metabolites generated in the periphery to diffuse into and condition the core. The deepening of the metabolic conditions sustaining the two different functional phenotypes of LSC is the object of current research in this speaker’s laboratory. The experimental strategy is based on the idea (well supported by previous findings) that the metabolic compartmentalization of CML cells, and LSC subsets, in particular, to occur in vivo in the function of space can be mimicked via the progressive exhaustion of nutrients (especially glucose) in vitro in the function of incubation time. This enables LSC subsets with different BCR/Ablprotein expression and metabolic proles to be studied separately, at different times of culture. Culture conditions are modulated by adding or subtracting metabolites or inhibitors and cultured cells are analyzed to determine the metabolic phenotype, the expression of stem cell markers, and the maintenance or loss of stem cell potential. Recent research has focused on the role of lactate and glutamine in the conditioning of LSC environment, as well as on the testing of drugs different from the TKi currently used for CML therapy. This research has been largely carried out using primary cell explanted from CML patients and murine CML models.

Dr. Fabio Sallustio (University of Bari, Bari, Italy) concluded the work of the second day of the conference by speaking about endothelial dysfunction of LPS-induced acute kidney injury (AKI). Endothelial cells (EC) acquire a fibroblast-like phenotype and contribute to myofibroblasts generation through the endothelial to mesenchymal transition (EndMT) process. Noteworthy, human renal stem and progenitor cells (ARPCs) enhance the tubular regenerative mechanism during AKI, but little is known about their effects on EC. Dr. Sallustio investigated whether ARPCs prevent sepsis-induced EndMT, and the related mechanism. When activated by LPS, EC proliferated and decreased specific EC markers, such as CD31 and VE-cadherin, and upregulated myofibroblast markers, such as collagen I and vimentin [17]. It was found that ARPCs normalized EC proliferation rate and abrogated the LPS-induced EndMT by restoring the high expression of EC markers and the low expression of myofibroblast markers. The gene expression analysis showed that most of the genes modulated in the LPS-stimulated ARPCs belong to cell activation and defense response pathways. It was demonstrated that the ARPC-specific anti-fibrotic effect is exerted by the secretion of CXCL6, SAA2, SAA4, and BPIFA2 and that three of the four chemokines were produced by ARPCs after the anaphylatoxin stimulation. For the first time, the SAA4 isoform was identified as a protein secreted by a renal cell, and specifically by renal progenitors, in response to LPS. Serum amyloid A (SAA) proteins belong to a family of an acute-phase protein whose biological functions are debated. In particular, SAA4 is poorly characterized and it has been recently found primarily induced in the liver during acute bacterial infection, in pigs. In this study, it was found that SAA4 is also released by human renal cells and, through an in vivo model of sepsis, in pig kidneys following LPS administration. It was demonstrated that this SAA isoform has a confined antiseptic function and can decrease local organ damage. The molecular signaling that underlies ARPC protective mechanism was also investigated, demonstrating that they diverge from RPTEC and from EC in MyD88-independent pathway activation following LPS stimulation, at least in the short time. Finally, a swine model of LPS-induced AKI was used to confirm in vivo the CXCL6, SAA4, and BPIFA2 secretion by ARPCs as a defense response.

The third conference day was opened by a new session entitled “Stem cells and cancer part-II”. The first speaker was Dr. Ilio Vitale (University of Roma Tor Vergata, Rome, Italy) who spoke about cancer stem cells (CSCs), a subpopulation of multipotent stem cells (SCs) responsible for the initiation, long-term clonal maintenance, and dissemination of most human neoplasms, and driving tumor relapse and resistance to conventional therapies [18]. CSCs reportedly share with embryonic and adult SCs a very robust DNA damage response (DDR), which favors the survival and resistance to genotoxic stress and can be exploited for therapeutic purposes. To elucidate the molecular mechanisms of the DDR in CSCs, a large panel of colorectal cancer (CRC) patient-derived tumorspheres enriched for CSCs (CRC-SCs) were generated. Through genetic, cytogenetic, and molecular analyses, it was demonstrated that CSCs have high but heterogeneous levels of replication stress (RS) in unperturbed growth conditions. Constitutive RS in CSCs is mainly fostered by p53 deficiency coupled with the presence of supernumerary chromosomes, an aneuploid condition known as hyperdiploidy. Moreover, evidence was provided that the replication stress response (RSR) is functional and plastic in CSCs, and its circuitry involves the coordinated activity of CHK1, PARP1, and components of the homologous recombination repair. Such an efficient RSR ensures tolerability to high constitutive RS to CSCs. It also confers to CSCs with elevated constitutive levels of RS a unique dependence on the functions of DDR component(s) such as CHK1, whose targeting can, thus, be an effective anti-CSC strategy. Nonetheless, RSR plasticity paradoxically favors the acquisition of resistance of CSCs to replication poisons or RSR inhibitors. Building on these findings, optimal RS-related anticancer protocols have been developed to eradicate and prevent resistance development in all CRC-SC subsets bearing distinct genetic backgrounds or ploidy profiles.

The next speaker was Dr. Carmela dell’Aversana (University of Campania “L. Vanvitelli”, Naples, Italy) who spoke about the epigenetic mechanisms that regulate the miRNA expression and, conversely, on the subsets of miRNAs that act as important epigenetic regulators, establishing a regulatory circuit to stabilize and modulate gene expression patterns [19]. The link between miRNA deregulation and malignancies has been clearly delucidated. Some reports identified “epidrug” responsive miRNAs whose expression was affected by “epi”treatment in different cancers. However, insights into miRNA function in cell behavior, differentiation, stemness, in self-renewal, cellular reprogramming and pathology, and their mode of action have so far been scarce. Dr. dell’Aversana identified and characterized the mechanism(s) by which miR-194-5p and its newly discovered target BCL2-associated transcription factor 1 (BCLAF1) regulate leukemogenesis. In acute myeloid leukemias (AML), the balance between miR-194-5p and BCLAF1 is perturbed, locking cells into an immature, potentially “immortal” state. Enhanced expression of miR-194-5p by treatment with the histone deacetylase inhibitor SAHA or by exogenous miR-194-5p expression resensitizes cells to differentiation and apoptosis by inducing differential compartmentalization of BCLAF1. The miR-194-5p/BCLAF1 balance was found commonly deregulated in 60 primary acute myeloid leukemia patients and was largely restored by ex vivo SAHA treatment. Moreover, miR-194-5p impacted cell fate leading myeloid differentiation toward fully specialized cells, in this case to dendritic cells. In conclusion, it was discovered that miR-194-5p/BCLAF1 equilibrium has an impact on maturation, cell fate, and susceptibility to anticancer treatment by new epigenetic setting and chromatin accessibility. The prospect microRNA-based reprogramming technology could have future applications in cancer medicine.

This session was opened for young researchers by Dr. Caterina Puglisi (IOM Ricerca, Viagrande, Italy) who spoke about cancer stem cells (CSCs) that have been identified in several solid tumors, including melanoma, breast, brain, lung, and colon cancer. CSCs are tumor cell subpopulations with indefinite proliferative potential that drives the growth of tumors. Nowadays, CSCs represent the main target of the recent therapeutic efforts to eradicate the malignancy, because they are capable of promoting metastatic dissemination and secondary resistance to treatments. Hence, the need to develop new therapeutic strategies aimed at specifically targeting the cancer stem cell population. Lung cancer is the leading cause of cancer mortality worldwide. Despite advances in anticancer therapies, the survival rates remain poor (<15%), thus suggesting that the development of new and more effective therapies are greatly needed. In this specific field, radiotherapy represents the first-line treatment for many inoperable lung tumors. During the last few years, a number of new technologies have been developed in the field of radiotherapy providing new opportunities for the treatment of lung cancer. To take full advantage of this increased availability, there is an increasing need for the personalization of therapy. Personalized radiotherapy is essential to effectively predict the response to therapeutic protocols in order to identify the most suitable cure for every patient. Dr. Puglisi has developed predictive in vitro and in vivo models, based on patient-derived CSCs, for the prediction of treatment effectiveness, thus, supporting the clinical decision in a personalized fashion. The objective of the model is the identification of personalized radiotherapy treatments, in terms of differential combinations of supplied doses and dose rates, in order to optimize the long-term response of patients with lung cancer and minimize undesired radiotoxic effects. CSCs have been isolated from tumor tissues of lung cancer patients and used to evaluate the effects of different protocols of radiotherapy in vitro. An evaluation of the effects on cell proliferation, apoptosis, and stem-cell-like phenotype has shown that some CSCs resulted sensitive at low radiating doses (8 Gy), whereas others showed a response only at high doses (10 and 20 Gy). Only one case, the treated CSCs, demonstrated complete resistance to all tested radiation treatments suggesting, in some cases, that radiotherapy would not be sufficient as a monotherapy. The molecular characterization of some checkpoint genes suggested a functional explanation of this increased radioresistance. The CSCs-derived animal models have also been evaluated in order to verify if the in vitro sensitivity to specific protocols is also maintained in vivo. Immunodeficient mice were transplanted using the patient-derived CSCs and treated (after the development of heterotopic tumors) using different protocols to evaluate the effects of a different combination of dose and dose rate as in vivo treatment. The preliminary data showed a significant relationship between the in vitro and in vivo sensitivity, suggesting that the cellular model could be sufficient to assess the suitability of treatment, and providing a feasible translational approach for the personalization of the cures. The use of radiation treatments designed to hit and destroy CSCs could change the way to treat cancer, reducing the risk of metastasis and of relapses and also reducing the risks and side effects of current treatments on healthy cells.

The last young researcher to speak was Dr. Michele D’Angelo (University of Aquila, L’Aquila, Italy) who reported on some recent studies that provided strong support for the cancer stem cell (CSC) hypothesis that suggests that many cancers, including breast cancer, are driven by a subpopulation of cells that display stem cell properties. CSCs represent a small population of cancer cells that exhibit self-renewal and differentiation characteristics similar to normal stem cells but differ in that their self-renewal pathways are deregulated. CSCs are responsible for tumor formation, progression, metastasis, and recurrence, as well as, drug resistance, based on the CSC presence. Thus, targeting CSCs may constitute a promising target for cancer prevention and treatment, especially in triple resistant breast cancer. Peroxisome Proliferator-Activated Receptors (PPARs) are steroid hormone receptors that, upon ligand activation, heterodimerize with the retinoid X receptor, binding to the specific promoter sequence (the peroxisome proliferator response element), triggering the expression of a variety of target genes, including those involved in glucose, lipid, and amino acid metabolism [20]. The PPAR receptors have a pivotal role in malignancy, although exerting pleiotropic effects, due to their multiple functions. In this study, mammospheres were isolated and characterized for the stemness markers and for the presence of PPARs. Since PPARα appeared as the most abundant isotype, cells were treated with a specific PPARα antagonist (GW6471). For this model, the effects on the energetic metabolism and cell proliferation and survival of breast cancer stem cells were investigated. PPARα inhibition determined a strong effect on cell viability and proliferation that could be related to the marked effects on metabolism. In fact, the inhibition of PPARα led to a decrease of cholesterol biosynthesis and of glucose uptake and metabolism, paralleled by an increase of lipid droplets consumption. Moreover, it was showed that an increase of CD36 protein levels, a fatty acid transporter, was observed upon GW6471 treatment, also indicating an imbalance of fatty acid uptake. It is worth noting that CD36 is a target gene of PPARγ and that GW6471 treatment resulted in an increase of PPARγ levels and its nuclear translocation. The metabolic reprogramming observed upon GW647 treatment was further confirmed by the increased protein levels of the AMP-activated protein kinase (AMPK), a cellular energy sensor that mediates metabolic homeostasis. Since mitochondria play a central role in tumor cell reprogramming, the function and the dynamic of this organelle were also investigated. Mitochondrial functions were not changed by PPARα antagonist treatment, while their cellular morphology and cellular localization were altered by treatment. Notably, control cells showed fused mitochondria particularly localized in the periphery of the cells, closeness to lamellipodia; upon treatment, the organelles lost fused morphology and resulted absent in the periphery of the stem cell lamellipodia. The presence of fused mitochondria in lamellipodia is necessary as energy support for cell migration, which suggests the existence of strongly migrating cells in control mammospheres and loss of this migrating ability after PPARα antagonist treatment. These results were further confirmed by the reduction in Rac, RhoA, and Cdc 42 levels upon antagonist treatment. Several lines of evidence indicate that the Rho family member Rac could play a critical role in different aspects of tumorigenesis such as the metastatic tumor ability.

The last session of the conference “Extrinsic signaling and stem cell functions” had, as a first speaker, Dr. Elisa Cimetta (University of Padua, Padua, Italy) who spoke about the miniaturization that has become a dominant trend in biomedical research, with engineers playing a crucial role in binding technology and biology [21]. In our bodies, cells reside in a complex milieu, the microenvironment (μEnv), that regulates their fate and function. Most of this complexity is lacking in standard laboratory models, leading to readouts that poorly predict the in vivo situation. This is particularly the case in cancer research, as tumors are extremely heterogeneous and capable of conditioning both the local μEnv and distant organs. Neuroblastoma (NB) is a heterogeneous pediatric malignancy of the sympathetic nervous system accounting for up to 10% of childhood cancers and with a strong tendency to metastasize. Secreted exosomes (EXO) are means by which NBs reshape their μEnv and induce local and long-range changes in cells, regulating progression and prognosis. The mechanisms involved are still not completely understood, and a major limitation is a difficulty to in vitro model the local in vivo dynamic μEnv. Among other factors, hypoxia is a key feature of solid tumors, specifically known to favor NB metastasis and dedifferentiation towards immature stem cell-like phenotypes and to stimulate EXO release, facilitating intercellular communication at distant sites. Dr. Cimetta proposed developing microfluidic bioreactors (μBRs) and testing their edge over classical approaches in decoding the role of EXO and the μEnv in NB. The μBRs generate time and space-resolved concentration gradients, support fast dynamic changes and reconstruct complex interactions between cells and tissues, while performing multifactorial and parallelized experiments. In this study, the proteomic and miRNAs cargo of EXO isolated from NB cell lines cultured at different oxygen concentrations were also characterized to identify an exosomal signature associated with NB metastatic dissemination. Exosomes were successfully isolated and purified from NB cell lines. Surface markers analysis revealed that NB hypoxia-derived EXO expressed an increase of proteins associated with angiogenesis, adhesion, stemness, and immune function. The proteomic cargo of EXO isolated from cultures in normal and hypoxic conditions were characterized and revealed the differential expression of about 90 proteins. The miRNA cargo profiles also highlighted significant differences between cell lines and within the different oxygenation conditions. Finally, first μBRs prototypes proved successful in generating stable concentration gradients over cultured cells.

The second speaker, Dr. Roberta Tasso (University of Perugia, Perugia, Italy), talked about mesenchymal stromal cells (MSCs) as effective therapeutic agents enhancing the repair of injured tissues. Preliminary results indicated that the paracrine activity of MSC promotes a functional switch of macrophages from a pro- (M1) to an anti-inflammatory (M2) state. Since extracellular vesicles (EVs) are relevant components of the MSC secretome, the aim of this study was to carry out a detailed characterization of EVs released by human adipose-derived MSCs to investigate their involvement as modulators of MSC anti-inflammatory effects [22]. The EV-isolation method was based on differential centrifugations of the medium conditioned by MSC exposed to either normoxic or hypoxic conditions (EVNormo and EVHypo). Both types of EVs were efficiently internalized by responding to bone marrow-derived macrophages, eliciting their switch from an M1 to an M2 phenotype. Observations that different macrophage subsets are associated with different stages of muscle regeneration led to an investigate to determine whether EV treatment could influence macrophage polarization from M1 to M2 phenotype in vivo. Taking advantage of a cardiotoxin (CTX) injury mouse model, a downregulation of IL6 and Nos2 was observed, concurrent to a significant upregulation of Arg1 and Ym1, late markers of alternative activation in injured and EV-treated muscles. These effects, accompanied by an accelerated expression of the myogenic markers Pax7, MyoD, and eMyhc, were even greater following EVHypo administration. Collectively, this data indicated that MSC-EVs possess effective anti-inflammatory properties, making them potential therapeutic agents that are handier and safer than MSCs.

The last session also provided time for young researchers to express their results. The first presenter of this session was Dr. Giulia Cricrì (University of Milan-Bicocca, Milan, Italy) who spoke about B-Cell precursor acute lymphoblastic leukemia (BCP-ALL), which is the result of a growing number of cooperating genetic and epigenetic aberrations that corrupt hematopoietic developmental pathways and lead to uncontrolled production of malignant B lymphoid precursor cells within the bone marrow (BM) niche. It remains the leading cause of childhood cancer-related death. In the BM niche, stromal and leukemic cells dialogue through soluble factors, cell-to-cell contact, and extracellular vesicles (EVs), which are increasingly recognized as modes of local, as well as distal, intercellular communications. In several hematological malignancies, such as acute myeloid and chronic lymphocytic leukemia, EVs reprogram the surrounding stroma to become leukemia-supporting and chemoprotective. Notably, EVs’ contribution in BCP-ALL pathogenesis has not been fully explored. In the context of BCP-ALL, Dr. Cricrì recently identified ActivinA, a TGF-β family member, which confers an aggressiveness advantage to leukemic cells by increasing their intracellular calcium content [23]. In view of the ActivinA property to positively regulate cytosolic calcium and actin polymerization, which are associated to EVs formation, secretion, and cargo sorting, it was evaluated whether this molecule is able to influence EVs release by leukemic cells. Therefore, a method was optimized to isolate and characterize EVs from BCP-ALL cells by using the human lymphoblastic leukemia bone marrow-derived 697 cell line cultured in serum-free medium for 24 to 48 h in the presence, or not, of ActivinA (50 ng/mL). Cell culture media were analyzed with nanoparticle tracking analysis (NTA) to determine the EVs concentration (particle/mL) and size distribution. Total EVs (30 to 700 nm), exosomes (30 to 150 nm), and microvesicles (151 to 700 nm) were classified. It was demonstrated that the 697 cell line induced the production of both EV populations. Notably, the ActivinA treatment increased the mean concentration of exosomes derived from 697 by about 60% (*p* < 0.008) and 30% (*p* < 0.016) at 24 h and 48 h, respectively, as compared with an unstimulated condition. The microvesicles mean concentration was nearly significant after 24 h of ActivinA stimulation (*p* = 0.055), whereas at 48 h there was an increase of about 30% (*p* < 0.016) as compared with an unstimulated condition. Preliminary data indicated that the expression levels of exosome markers were higher as compared with that of microvesicles, thus confirming the results obtained with NTA software. Interestingly, it was observed that ActivinA induced an increased percentage of EVs expressing CD63 and CD9, respectively, 60% at 24 h and 45% at 48 h. EVs preparations were also used to quantify the total amount of proteins through a BCA assay. The average EV protein content was lower as compared with that of 697 cell line, without any difference between treated or untreated conditions. Regarding the untreated condition, at 24 h, the mean of total protein concentration was 29,392.0 μg in the cell and 6094.3 μg in the EV compartment, while, at 48 h, it was 33,007.7 μg and 6862.2 μg, respectively. Overall, it was demonstrated that ActivinA positively regulates BCP-ALL cell vesiculation. A better understanding of EVs’ role in the leukemia-stromal crosstalk could provide new therapeutic targets that could allow eradication of leukemic cells without adversely affecting healthy hematopoiesis.

The last speaker of the conference was Dr. Luigi Alfano (CROM, Mercogliano, Italy) who talked about the genomic instability as one of the enabling characteristics underlying tumor development. Many sources of DNA damage, both endogenous and environmental, affect genome integrity predisposing to cancer development. Stem cells (SCs), which are crucial for the generation and maintenance of intracellular heterogeneity, can be affected by many sources of DNA damage favoring the tissue and organism homeostasis alteration. The DNA repair pathways in the SCs can preserve the integrity of healthy cells, but also can be involved in the resistance to treatment of cancer stem cells (CSCs). The characterization of the DNA repair mechanisms can be helpful to improve cancer therapy both for cancer cells and CSCs. Dr. Alfano described a novel role for the heterogeneous ribonucleoprotein D (HNRNPD) in the regulation of DNA end resection in HeLa cells [24]. He performed a proteomic-screening assay to identify novel proteins involved in the DNA end resection. By annealing two complementary DNAs with a 40 nt protruding end at 5′ termini and a 3′-biotin tag on the bottom strand, a ssDNA/dsDNA structure was generated mimicking an intermediate of the first steps of DNA resection. By bioinformatics analysis, HNRNPD was identified as a putative DNA repair protein and the involvement of HNRNPD in the regulation the R-loop formation near the DNA damage lesions was demonstrated. The HNRNPD downregulation, through the siRNAs, in HeLa cells, impairs the DNA end resection measured by Western blot analysis of RPA S4/8. By FACS analysis of BrdU levels, ssDNA formation, in non-denaturing condition, was reduced; moreover, the generation of HeLa ER-AsiSI cell lines demonstrated an impairment in the resection of silenced HNRNPD cells. A HeLa HNRNPD knockout was generated through the CRISPR-Cas9 system showing RPA S4/8 reduction in response to CPT treatment; the transfection of HNRNPD P AM resistant plasmid rescued the RP A phosphorylation defects. It was demonstrated that HNRNPD interacts with the SAF-A (HNRNPU), another RNA binding protein involved in the regulation of R-loop resolution near the DSBs, favoring its localization to damaged sites. Given the role of SAF-A in the regulation of R-loop dynamic, it was shown that RNase H1 expression in HNRNPD KO cells was able to recover RPA32 S4/8 phosphorylation defects, further supporting the idea that HNRNPD acts favoring R-loop removal for a proper DNA end resection. Finally, impaired colony formation ability in HNRNPD KO cells was shown with respect to the wild-type control, both treated with Olaparib, suggesting a possible use of HNRNPD as a synthetic lethal approach.

## 4. Conclusions

Taken together, all these findings and results indicate several promising approaches for the future of stem cell biology research and potential clinical applications. The conference overall direction indicates that stem cell biology research requires further development in order to achieve a better understanding of the specific cell cycles controlling differentiation capacity, secretome activity, and host-integration processes before potentially translating into clinical applications. This matter also requires better funding assessment by granting agencies and governments. By a “bench” point of view, the future of stem cell research seems to be a better characterization of the overall secretome capacity of stem cells. It appears that the positive effects provided by stem cell transplantation could be mainly due to the huge molecular machinery secreted by stem cells. Specific and precise characterization of these biomolecules could provide better approaches for stem cell-mediated therapies. The clinical applications of stem cells indicate the need for disease-specific protocols. Administration routes, cell dose, timing, and uniformity of outcomes must be considered and finalized in order to provide optimal responses to the cell therapy.

The next conference is scheduled in June 2021, in Genoa, Italy.
